# Ferri–Liposomes: Preformulation and Selective Cytotoxicity against A549 Lung Cancer Cells

**DOI:** 10.3390/pharmaceutics13050712

**Published:** 2021-05-13

**Authors:** Marina Guedes Fonseca de Souza, Fabrícia Nunes de Jesus Guedes, Marli Luiza Tebaldi, Éverton do Nascimento Alencar, Lucas Amaral-Machado, Eryvaldo Sócrates Tabosa do Egito, André Luis Branco de Barros, Daniel Crístian Ferreira Soares

**Affiliations:** 1 Bioengineering Laboratory, Federal University of Itajubá, Rua Irmã Ivone Drumond, 200, Distrito Industrial II, Itabira 35903-087, Brazil; marinagueds@gmail.com (M.G.F.d.S.); fabriciajesus@gmail.com (F.N.d.J.G.); marlitebaldi@unifei.edu.br (M.L.T.); 2 Laboratory of Dispersed Systems (LaSiD), Department of Pharmacy, Federal University of Rio Grande do Norte (UFRN), Rua Gal. Gustavo Cordeiro de Farias, s/n, Petropolis, Natal 59010-180, Brazil; evertonalencar@ufrn.edu.br (É.d.N.A.); machado.lucasam@gmail.com (L.A.-M.); socratesegito@gmail.com (E.S.T.d.E.); 3 Faculty of Pharmacy, Federal University of Minas Gerais, Avenida Presidente Antônio Carlos, 6627, Pampulha, Belo Horizonte 31270-901, Brazil; albb@ufmg.br

**Keywords:** iron oxide liposomes, A549 cells, lung cancer treatment, selective therapy

## Abstract

Liposomes have become successful nanostructured systems used in clinical practices. These vesicles are able to carry important drug loadings with noteworthy stability. The aim of this work was to develop iron oxide-loaded stealth liposomes as a prospective alternative for the treatment of lung cancer. In this study, citric acid iron oxide nanoparticles (IONPs-Ac) were synthesized and encapsulated in stealth liposomes. Their cytotoxicity and selectivity against lung tumor cells were assessed. Stealth liposomal vesicles, with relevant content of IONPs-Ac, named ferri–liposomes (SL-IONPs-Ac), were produced with an average size of 200 nm. They displayed important cytotoxicity in a human lung cancer cells model (A549 cells), even at low concentrations, whereas free IONPs-Ac displayed adequate biocompatibility. Nevertheless, the treatment at the same concentration of ferri–liposomes against HEK-293 cells, a normal human cell lineage, was not significantly cytotoxic, revealing a probable lung tumor selectiveness of the fabricated formulation. Furthermore, from the flow cytometry studies, it was possible to infer that ferri–liposomes were able to induce A549 tumor cells death through apoptosis/ferroptosis processes, evidenced by a significant reduction of the mitochondrial membrane potential.

## 1. Introduction

Iron oxide nanoparticles are biocompatible nanostructured systems with noteworthy chemical stability. They have drawn attention in the scientific field due to their remarkable ability to act not only as a contrast agent in magnetic resonance imaging (MRI) procedures, for both longitudinal (T1) and transverse (T2) relaxation times, and magnetic hyperthermia techniques, but also as a drug delivery system [1,2,3,4,5,6]. Although presenting remarkable effectiveness, these systems revealed some technical drawbacks, among which their particle size stands out. This physicochemical parameter has remarkable importance, because optimum superparamagnetic properties are only observed in nanoparticles smaller than 20 nm, impairing its use in several diseases, such as cancer [5].

In fact, this range of particle size induces a great elimination clearance of the particles by the reticuloendothelial system. This leads to unsatisfactory tissue accumulation at the target site and reduces the efficacy of iron oxide nanoparticles in cancer treatment.

Several approaches have been used in order to overcome these drawbacks. Surface PEGylation or immobilization with folic acid and other targeting molecules have been adopted. Alternatively, ion-based liposomes have been also developed and showed promising applicability in cancer hyperthermia research [7,8,9]. In fact, liposomes are one the most successful nanostructured systems used in clinical practices. In addition to the ability to load large amounts of ions and drugs, liposomes are described as versatile biocompatible delivery systems and have been used in different therapeutic approaches [10,11,12].

Based on this rationale, studies performed by Matsuoka and colleagues (2004) and Peller and colleagues (2016) have described the anticancer efficacy of iron-based liposomes. These authors revealed, using an in vivo experimental approach, that ferri–liposomes can increase the temperatures of the tumor, leading to cancer cells death [13,14].

The main mechanism behind the cancer treatment with such carriers relies on the enhanced permeability and retention (EPR) effect [7]. Cancer can be characterized by the presence of cells with DNA changes that are able to quickly multiply themselves and stimulate angiogenesis in order to nourish the tumor. In this regard, because tumor-fenestrated capillary pores present an average diameter of 400 nm, nanostructured iron oxide-loaded carriers may permeate through the capillaries and, consequently, improve the tissue accumulation at the target site due to the EPR effect [7].

Indeed, after permeation into the cancer cells, this nanostructured system stimulates the Fe^2+^ions production, leading to a reactive oxygen species (ROS) overproduction. Consequently, the high ROS levels promote mitochondrial dysfunctions, inducing the cell’s death, triggered by apoptotic and/or necrotic mechanisms [15,16]. Moreover, the aforementioned reports also observed that these responses are significantly dose- and particle type-dependent, once different results were observed in different breast cancers and endothelial cells, suggesting that ion-based liposomes are promising nanostructured systems for medical applications [17]. Although showing remarkable activity against breast cancer and endothelial cells, these ion-based liposomes have not yet been evaluated against other tumors, such as lung cancer.

Lung cancer is the leading cause of cancer-related deaths in men and the second leading cause for women, worldwide. It has been estimated that this disease displayed more than 1.5 million new cases and deaths in 2012, alone, representing approximately 20% of all global cancer deaths [18]. Therefore, several therapeutic strategies have been developed in this field. However, the current marketed medicines present, in addition to several site effects (which significantly compromise the patient compliance), no specific targeting to cancer cells, leading to a high toxicity degree and drug resistance [19].

In light of this evidence, it is possible to highlight the importance of novel anticancer therapeutic approaches, such as the use of ion-based liposomes, for prospect lung cancer therapy. Therefore, the aim of this work was to develop ion-loaded stealth liposomes targeted to lung tumor cells as a potential alternative for treatment of lung cancer.

## 2. Materials and Methods 

### 2.1. Materials 

Ferrous sulfate heptahydrate (FeSO_4_·7H_2_O) and ammonium hydroxide (NH_4_OH, 27% (*w*/*v*)) were provided by Sigma–Aldrich (São Paulo, Brazil). Iron chloride hexahydrate (FeCl_3_·6H_2_O) and citric acid were provided by Dinâmica Química (Indaiatuba, Brazil). Ammonium ferric sulfate dodecahydrate (Fe(NH_4_) (SO_4_)_2_·12H_2_O) (99% (*w*/*v*)) was purchased from Merck (São Paulo, Brazil). Ultrapure water produced from a Smart2Pure 3 UV (ThermoScientific, Bremen, Germany) was used for all experiments. Dipalmitoylphosphatidylcholine (DPPC), distearoylphosphatidylcholine (DSPC), and distearoylphosphatidylethanolamine-polyethyleneglycol2000 (DSPE-PEG2000) were from Lipoid GmbH (Ludwigshafen, Germany). Chloroform was acquired from LabSynth (São Paulo, Brazil). HEPES (4-(2-hydroxyethyl)-1-piperazine-ethanesulfonic acid) was supplied by Sigma Chemical Company (St. Louis, MO, USA). BD Horizon™; BD Horizon Fixable Viable Stain 450 reagent and BD Horizon™ CFSE (carboxyfluorescein diacetate succinimidyl ester) were purchased from BD (São Paulo, Brazil). All chemicals were of analytical grade and used without further purification.

### 2.2. Synthesis and Functionalization of Ions Particles (IONPs-Ac)

IONPs were synthesized based on the previous study conducted by Racuciu et al. (2006), with some modifications [20]. Briefly, 1.12 g of FeSO_4_·7H_2_O and 2.16 g of FeCl_3_·6H_2_O were dissolved in 60 mL of ultrapure water. Subsequently, 40 mL of NH_4_OH (27% (*w*/*v*)) was added to the mixture at a flow rate of 10 mL·s^−1^ under mechanical stirring (Fisatom 713, São Paulo, Brazil) at 40 °C and 4000 rpm. Next, the system was heated and maintained at 60 °C for 30 min. The produced material was washed 8 times with ultrapure water, reaching a neutral pH. Finally, the obtained nanoparticles were functionalized with citric acid, by adding 1.0 mL of the organic acid (16.67 mg·mL^−1^). The system was maintained under magnetic stirring at 85 °C and 120 rpm for 90 min. The produced functionalized nanoparticles (IONPs-Ac) were washed with ultrapure water through centrifugation, at room temperature (25,000× *g*, 10 min) using an Amicon^®^ Ultra-4 (10 kDa MWCO, Millipore, Billerica, MA, USA, EUA) apparatus until the obtaining of a dispersion with neutral pH.

### 2.3. Ferri–Liposomes (SL-IONPs-Ac) Production

IONPs-loaded stealth liposomes (SL-IONPs-Ac) were prepared by the reverse phase evaporation technique following procedures previously developed by our research group [21]. A fresh formulation was prepared for each independent experiment to avoid stability-related errors during the experiments. The formulation was prepared from chloroform aliquots of DPPC, DSPC, and DSPE-PEG_2000_ in lipid molar ratio of 80:15:5 (a total of 40 mmol·L^−1^ of lipids content). The organic solvent was evaporated using an IKA Labortechnik HB4 rotary evaporator (Staufen, Germany) until a lipid film was obtained. Subsequently, the film was dissolved in diethyl ether (10 mL), previously treated with a solution of HEPES buffer at 10 mmol·L^−1^. After the complete dissolution of the lipids, an aqueous suspension of IONPs-Ac at 1840 µg·mL^−1^ was added to maintain an aqueous:organic suspension phase ratio of 1:3. Then, the achieved mixture was vortexed for 5 min (at room temperature) in order to produce a water in oil (W/O) emulsion, following the production of large unilamellar vesicles by diethyl ether elimination using the rotary evaporator. Finally, the produced liposomes were calibrated using 5 cycles of extrusion (Extruder T 001, Lipex Biomembranes, Vancouver, BC, Canada) on polycarbonate membranes of 0.4 and 0.2 µm pore sizes, under nitrogen pressure. The possible presence of precipitates and agglomerates after the extrusion process was evaluated by DLS analysis (Section 2.5).

Blank liposomes (without IONPs-Ac) were produced by the same experimental protocol, except for the step of addition of the nanoparticles suspension, which was replaced by the addition of 10 mmol·L^−1^ HEPES buffer. A total of 3 independent experiments were performed.

### 2.4. Physical and Chemical Characterization—X-ray Diffractometry and Fourier Transform Infrared Spectroscopy

In order to evaluate the IONPs crystallinity profile, samples were characterized by X-ray diffractometry. The analyses were conducted using a Rigaku model Geigerflex equipment (Tokyo, Japan) and an interval from 10 to 70° (2θ) and a rate of 4° per minute.

Fourier transform infrared spectroscopy (FTIR) was performed in order to characterize the typical chemical bonds of the samples. An ATR spectrophotometer (PerkinElmer, Model Frontier, Norwalk, CT, USA) was used, and analyses were conducted at room temperature. The samples were uniformly placed in a ZnSe crystal, and the spectra was recorded from 4000 to 400 cm^−1^, with resolution of 4 cm^−1^ and 20 scans. The experiment was performed in triplicate.

### 2.5. Physicochemical Characterization—Particle Size Distribution Evaluation

The average diameter and the polydispersity index (PDI) of the IONPs and vesicles were determined by dynamic light scattering (DLS) using a Nano ZS 90 Zetasizer (Malvern Instruments, Malvern, England) at 25 °C and fixed angle at 90°. The measurements were performed in triplicate. The samples were previously diluted in HEPES buffer (ratio at 1:20 (*v*/*v*)) The results were expressed as the mean ± standard deviation (SD) of three different batches of each formulation.

### 2.6. Encapsulation Efficiency Study

The encapsulation efficiency was determined using a method previously developed by Breitkreitz et al. (2014), with modifications. It allowed us to quantify the iron content of a solution through a complexation reaction between iron(II) ions and the chelation agent, Phenanthroline (Ph) [22]. Initially, a standard solution of Fe–Ph (4000 μg/mL) was prepared by a complexation reaction between O-phenanthroline and Fe(NH_4_)(SO_4_)_2_·12H_2_O (50 mg·mL^−1^), in an equal molar ratio. Ascorbic acid was used as a reducing agent. The maximum absorbance of the Fe–Ph complex was determined by UV/Vis spectrometry wavescan from 400 to 600 nm (*n* = 3) (UV-2600 Shimadzu, Tokyo, Japan). Subsequently, 6 different standards ranging from 0.5 to 5.0 μg/mL of Fe–Ph were prepared to construct a calibration curve.

In order to separate the non-entrapped IONPs-Ac from the vesicles, SL-IONPs-Ac formulations were previously purified by centrifugation (Megafuge 16R ThermoScientific, Darmstadt, Germany) at 15,000× *g* and 4 °C for 15 min. The supernatant was removed, and the pellet was resuspended using purified water (1 mL). The encapsulation efficiency evaluation was performed using the aforementioned method, in which the replicates of purified SL-IONPs-Ac samples (1 mL) were placed into 15 mL polystyrene tubes containing 5 mL of isopropyl alcohol (HPLC grade) in order to promote the prompt release of IONPs-Ac from the vesicles. Posteriorly, 1 mL of a solution of O-phenanthroline (6 mg·mL^−1^) and 1 mL of ascorbic acid (6 mg·mL^−1^) were added, homogenized, and then the encapsulated iron oxide into the liposomes was quantified (wavelength set at 510.6 nm). The supernatant obtained in the purification process, containing the non-entrapped nanoparticles, was also used to determine, indirectly, the encapsulation efficiency of the vesicles and, thus, to confirm/corroborate the obtained data. All analyses were performed in triplicate.

### 2.7. Physicochemical Stability Evaluation

The SL-IONPs-Ac sample was stored at 10 ± 2 °C, and its particle size distribution was monitored over 31 days in triplicate. Liposomes aliquots (50 μL) were diluted with saline solution (1:20 *v*/*v*), and the analyses were performed as described in the physicochemical characterization section.

### 2.8. In Vitro Biological Evaluation

#### 2.8.1. Cytotoxicity Evaluation

To investigate the cytotoxicity profile of the IONPs-Ac and SL-IONPs-Ac, two human cell lines were used (an embryonic kidney cell model—HEK-293-ATCC^®^ CRL-1573—and a lung adenocarcinoma cell model—A549-ATCC^®^ CCL-185). The cell lines were cultured in appropriate cell culture flasks of 50 mL. Eagle modified by Dulbecco (DMEM) (4500 mg·L^−1^ glucose; 4 M; glutamine; 11 mg·L^−1^; sodium pyruvate; 3.7 g·L^−1^ sodium bicarbonate) (Sigma–Aldrich, São Paulo, Brazil) supplemented with fetal bovine serum at 10% and streptomycin 1% was used as the culture medium. The cells were incubated at 37 °C in a CO_2_ incubator model 311 (Thermo Fisher Scientific, Asheville, NC, USA) with controlled atmosphere (95% O_2_; 5% CO_2_) and humidity. After suitable confluence, approximately 1.0 × 10^8^ cells were transferred to cell culture plates with 12 wells, in which they remained for 24 h to properly adhere to the ground of the plate.

The samples were dispersed in PBS solution and incubated into the culture plate wells. This procedure was performed for each cell line. The following experimental groups were established (*n* = 2/group): (i) sterile NaCl solution (0.9% (*w*/*v*)) as negative control, (ii) DMSO solution (20%) as positive control, (iii) IONPs-Ac (150 μg·mL^−1^), (iv) blank liposomes (total lipid concentration of 40 mM), (v) SL-IONPs-Ac (3.8 μg·mL^−1^), (vi) SL-IONPs-Ac (1.9 μg·mL^−1^), and (vii) SL-IONPs-Ac (0.9 μg·mL^−1^). All experiments from cell culture and flow cytometry assays were carried out following the biosecurity standards described by the ISO 10993-5 (2009). All used materials were previously sterilized, and all step processes of cell manipulation were performed in a biological safety cabinet BIOSEG 12, Class II type A1 (Veco Group, São Paulo, Brazil).

Flow cytometry analysis was performed using the reagent kit Fixable Viability Stain^®^ 450 (V450, BD Biosciences, São Paulo, Brazil), which was able to discriminate living cells from unviable ones. For each sample, 50,000 events were acquired to perform the appropriate statistical treatment.

The cells’ morphology was evaluated using an Olympus BX43 light microscope equipped with a DP-73 camera (Olympus, Shanghai, China). Blue trypan was used as a stain, and the assays were performed in triplicate.

#### 2.8.2. Cell Death Mechanism Evaluation

The cell death mechanism was evaluated in A549 cell line through the use of FITC annexin V apoptosis detection kit and the 7-amino-actinomycin dye (7-AAD, BD Biosciences, São Paulo, Brazil). Cells were seeded into a 12-well plate (2.0 × 10^5^ cells/well) and treated with SL-IONPs-Ac for 48 h (iron concentration of 3.8 µg·mL^−1^). Next, cells were washed (2×) with PBS solution and resuspended with 400 µL of a stain buffer (Pharmingen, BD Biosciences, São Paulo, Brazil). Subsequently, the cells were incubated in a dark environment with 5 µL of FITC annexin V apoptosis detection kit for 15 min and stained with 10 µL of 7AAD dye for an additional 5 min. Finally, the cells were washed with PBS buffer solution (2×) and analyzed by flow cytometry (FacsVerse, BD, São José, CA, USA). All procedures were conducted at 10 °C. Cells treated with PBS solution and 12 µM of camptothecin (pharmaceutical standard, Sigma–Aldrich, São Paulo, Brazil) were used as negative and positive control, respectively.

#### 2.8.3. Mitochondrial Membrane Potential Evaluation

The mitochondrial membrane potential (∆Ψ_m_) was assessed by the tetramethyl–rhodamine ethyl ester (TMRE) (MitoStatus-BD Biosciences, São Paulo, Brazil) reagent. A549 cells were treated with SL-IONPs-Ac at 3.8 μg·mL^−1^ (*n* = 8) for 48 h. A total of 50 mM of a freshly prepared stock solution of TMRE was added to 3.0 × 10^6^ cells (*n* = 8). The cell suspensions were transferred to 15 mL polypropylene tubes and incubated for 25 min at 37 °C, protected from the light. Subsequently, the samples were centrifuged (1800 RPM, 3 min, room temperature) and washed using PBS. The flow cytometry analyses were used to evaluate the ΔΨ_m_ from a total of 50.000 events.

### 2.9. Statistical Analysis

Data of liposome characterization were evaluated through ANOVA test, followed by Tukey’s test. Data of the cytotoxicity study were evaluated by ANOVA test, followed by Bonferroni’s test. For all analyses, differences were considered significant when the p-value was lower than 0.05. All statistical analyses were performed using the Prism 5.0 software (GraphPad Software Inc., La Jolla, CA, USA).

## 3. Results and Discussion

In order to ensure the successful production of the formulation, the visual characterization of the IONPs, IONPs-Ac, and SL-IONPs-Ac was performed. The synthesized IONPs were obtained as a fine black-dark powder. The presence of citric acid had a fundamental role in the dispersion of the iron oxide nanoparticles: once, it was observed that a uniform dispersion without precipitates and agglomerates was maintained, even after 60 days. Indeed, the high stability of the system can be attributed to the presence of citric acid molecules covalently linked to IONPs nanoparticles. Furthermore, the iron oxide nanoparticles-loaded liposomes (SL-IONPs-Ac) presented as a milky-aspect dispersion, like blank liposomes (control), differing only by the light-brown color due to the IONPs-Ac presence. In addition, a single uniform phase was observed, indicating a homogeneous production of the system.

### 3.1. Physical and Chemical Characterization

**Figure 1:**
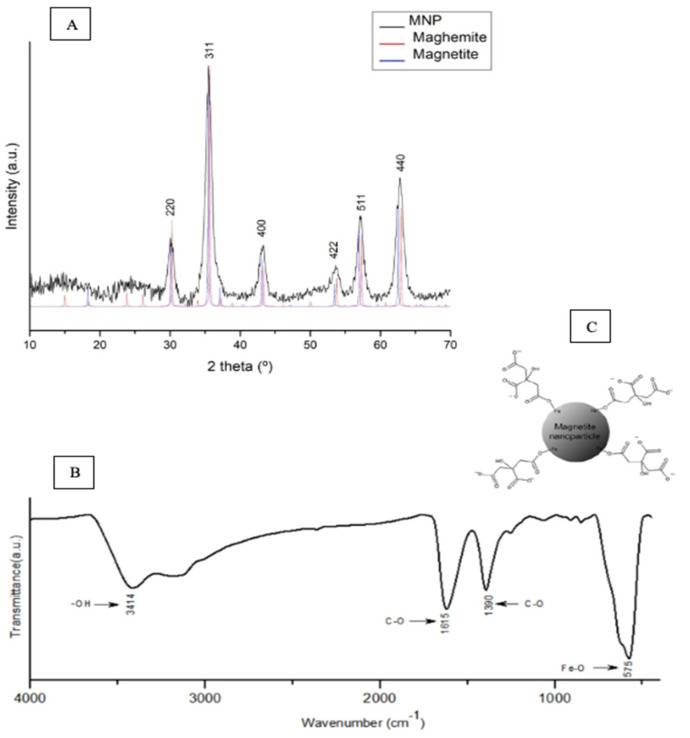
(**A**) XRD patterns of nonfunctionalized IONPs samples. The main peaks were compared to the powder diffraction files of magnetite (19-1629) and maghemite (39-1346). (**B**) FTIR spectrum of IONPs-Ac samples obtained from 4000–550 cm^−1^. (**C**) Suggested model of the obtained IONPs-Ac.

The XRD diffractogram of the IONPs samples (Figure 1A) revealed six main peaks, which were attributed to the following crystalline planes (220), (311), (400), (422), (511), and (440). This investigation was conducted in samples without citric acid functionalization. The XRD patterns were compared to the standards from the powder diffraction files (PDF), which corroborated the files 19-1629 and 39-1346 from magnetite and maghemite crystals, respectively. The average crystallite size was calculated by the Scherrer’s equation, and the full width at half maximum (FWHM) value was determined through the Origin Software (OriginLab, Northampton, MA, USA).

FTIR analyses were performed in order to evaluate the citric acid functionalization in samples of IONPs-Ac, which presented a band at 1390 cm^−1^ (Figure 1B), attributed to the asymmetric stretch of C-O bond. FTIR of the pure citric acid revealed a stretch of the C=O bond at 1700 cm^−1^. However, in Figure 1B, a slight offset of this band can be observed, since the stretch of the C=O bond was visualized at 1615 cm^−1^. This data can be explained by the fact that the carboxylate group is linked to the surface of the iron oxide nanoparticles, which promoted the C=O band shift [23]. In addition, a strong and wide band at 3414 cm^−1^ was attributed to O-H stretch, which might be related to the surface water absorption and the hydroxyl groups of the citric acid [24], as suggested in Figure 1C. Finally, the band at 575 cm^−1^ was attributed to Fe-O bonds.

The data of the average hydrodynamic size and polydispersity index of IONPs-Ac and SL-IONPs-Ac samples (Table 1) were determined through dynamic laser scattering (DLS) technique. The obtained data revealed that small and polydisperse IONPs nanoparticles, reaching approximately 13 nm, were obtained through the proposed synthetic route. On the other hand, monodisperse liposomes with size of around 200 nm, loaded with IONPs, were produced. Such results are in agreement with the work of Lu et al. 2019, who developed similar systems, wherein Camptosar (CPT-11) was co-encapsulated with citric acid-coated magnetic Fe_3_O_4_ nanoparticles and the formulation was surface-conjugated with Cetuximab. Their results revealed a particle size of around 180 nm, showing that our method is in agreement with the magnetic liposomes’ researched formulations.

### 3.2. Encapsulation Efficiency Study

The encapsulation efficiency was quantified by UV/Vis spectroscopy technique (UV-2600 Shimadzu, Tokyo, Japan). The photometric scanning of the iron(II)–Phenanthroline (Fe–Ph) complex revealed that a maximum absorbance was obtained at 510.6 nm. Thereafter, the calibration curve was obtained using six different standards ranging from 0.5 to 5.0 µg·mL^−1^ of Fe–Ph, and the spectrophotometer was set up at 510.6 nm.

The IONPs-Ac encapsulation efficiency in the liposomal vesicles was calculated from the absorbance results obtained in triplicates, considering the dilution process. A total of 228.5 ± 9 µg·mL^−1^ of iron was found on the IONPs-Ac, which corresponds to an encapsulation efficiency of 12.45 ± 0.76%. Similar data was reported by Lu et al. (2019), who developed thermosensitive magnetic liposomes to be applied to brain tumor chemotherapy and showed an encapsulation efficiency of 9.7 ± 1.4% [25]. In the mentioned study, the authors prepared the liposomes with a suspension of iron oxide nanoparticles at 1000 µg·mL^−1^, whereas in our study, a suspension of IONPs-Ac was prepared at 1840 µg·mL^−1^, which allowed a reasonable increase in the encapsulation efficiency.

### 3.3. Preliminary Formulation Stability

**Figure 2:**
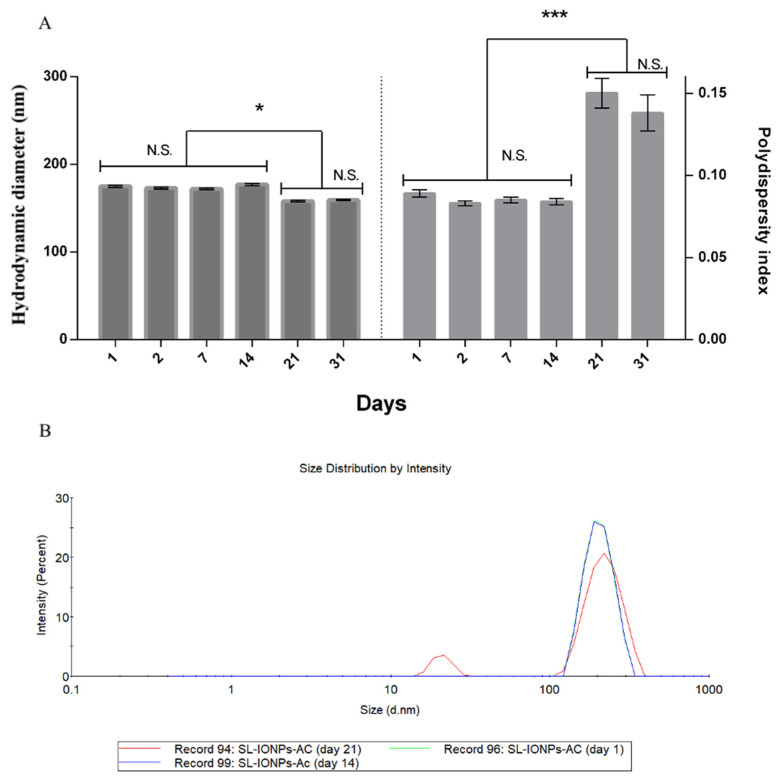
SL-IONPs-Ac storage stability study conducted at 10 °C for 31 days. (**A**) Left panel: average hydrodynamic diameter values; Right panel: PDI values. (mean ± S.D). N.S. = non-statistically significant differences; * (*p* < 0.5); *** (*p* < 0.01). (**B**) Size distribution of SL-IONPs-Ac in terms of intensity after 1, 14, and 21 days. Data obtained after 1 and 14 days are mostly overlapped (blue and green lines).

The stability of the liposome formulation (SL-IONPs-Ac) was evaluated for the samples stored at 10 °C for 31 days (Figure 2A). The results revealed a suitable stability of the vesicles, since the average hydrodynamic diameter remained stable over 14 days, in which the PdI values did not present statistically significant differences (*p* < 0.01). Nonetheless, it is important to note a slight, but not statistically different, decrease in PdI between day 1 and day 2. This can be explained by the Ostwald ripening phenomenon, normally observed in the first 24 h after the production of the dispersed systems. Ostwald ripening can be described by the incorporation of free molecules or smaller particles/droplets to higher ones, resulting in changes (mostly decreases) in PdI values [26]. In addition, after 21 days, it was possible to verify a significant increase in the PdI values (from 0.083 to 0.151) and a reduction in the average hydrodynamic diameter of the liposome (from 189 to 179 nm) (*p* < 0.05). This behavior is suggestive of IONP-Ac release from SL-IONPs-Ac, since two populations of particles were observed, ranging from 20–25 nm and 200–230 nm, attributed to the IONPS-Ac samples and liposomes, respectively (Figure 2B). After 31 days, no significant change was observed in the system, compared to the change that occurred after 21 days. Further investigations still need to be conducted regarding the release profile of this formulation and how it could affect the formulation stability and pharmacological parameters in vitro and in vivo.

It is important to highlight that, throughout the storage stability study, the PdI was below 0.3, which ensures a homogeneous distribution of the vesicles. Based on these results, the physicochemical characterization remained under acceptable scientific criteria. Hence, it is possible to affirm that the SL-IONPs-Ac was successfully produced and presented suitable particle size distribution, morphological features, and stability.

### 3.4. Cytotoxicity and Selectivity Evaluation

**Figure 3:**
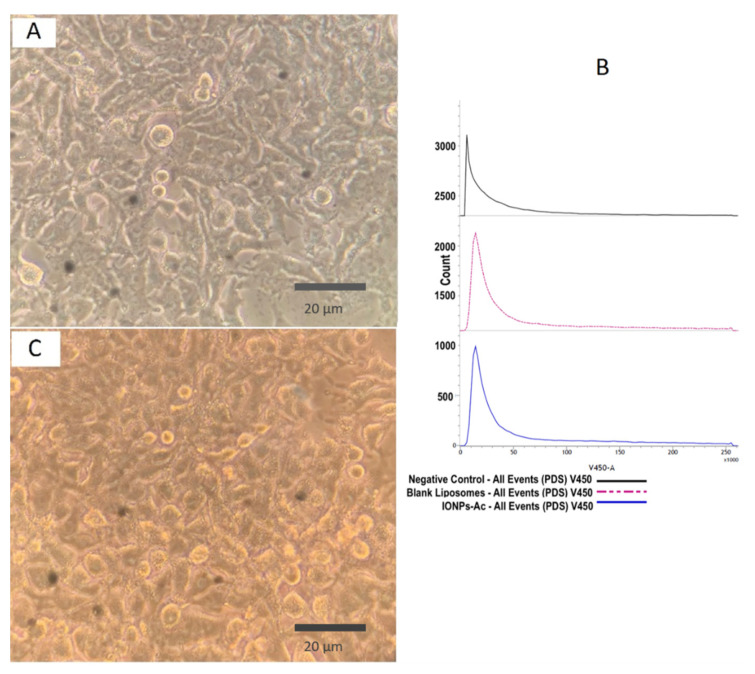
Micrographs and flow cytometry panel obtained from A549 cells: (**A**) Negative control (cells treated with NaCl 0.9% (*w*/*v*))—2000×). The cells were treated with IONPs at 150 µg·mL^−1^, and each chart represents (**B**) a histogram of Fixable Viable Stain 450 (V450-A) fluorescence intensity revealing the majority one single population, which was attributed to healthy cells. Data obtained from the count of 50,000 events. (**C**) Micrograph of treated A549 cells in amplification of 2000×.

Initially, the cytotoxicity of samples, IONPs-Ac (150 μg·mL^−1^) and blank liposomes was evaluated in A549 tumor cells through the flow cytometry technique and optical microscopy. Figure 3A shows the micrograph of A549 cells treated with NaCl solution (0.9% (*w*/*v*)) used as a negative control and the parameter for the flow cytometry gating (amplification of 900×). The cells were treated with IONPs-Ac for 48 h, and the results revealed that, even at high concentrations of IONPs-Ac, only a single population of living cells was detected, which is characterized by the peak in the flow cytometry histogram of Fixable Viable Stain-450 (V-450) emissions (Figure 3B). Similar data can be also observed in Figure 3C (amplification of 2000×), which represents A549 cells treated with blank liposomes, allowing the suggestion of the absence of relevant cytotoxicity.

The performed assay used the V450-A, a staining compound that has a maximum emission at 450 nm. This molecule binds, covalently, with amine radicals of the cell’s surface and, in minor quantities, in the intracellular medium, allowing us to observe the fluorescence levels from the cells. Indeed, the fluorescence evidenced by this assay allowed us to correlate the presence of dead and live cells, once dead cells ones showed increased fluorescence compared to living cells. Based on this rationale, it is possible to suggest that the low fluorescence level, observed in the Figure 3B, is an indicative of living cells.

Iron oxide nanoparticles have drawn the attention of researchers due to their biomedical applications, magnetic properties, and remarkable biocompatibility [27,28,29,30]. However, limited studies using iron oxide nanoparticles, specifically as a potential agent against cancer, have been disclosed. Watanabe et al. (2013) studied the cytotoxicity of magnetic iron oxide nanoparticles (size ranging from 200–450 nm) against A549 tumor cells and showed minimal effect in cells viability, at concentrations up to 100 µg·mL^−1^. At this concentration, a small percentage of cells (2.5% of the cells) underwent oxidative damage and apoptosis [31]. Therefore, the overall data of the mentioned study corroborates our findings, wherein no significant effects were observed in the A549 cells treated with IONPs-Ac at 150 μg·mL^−1^.

A549 tumor cells were also treated with SL-IONPs-Ac (iron oxide concentrations at 3.8 µg·mL^−1^, 1.9, and 0.9 µg·mL^−1^) for 48 h. The results revealed a dose-dependent cytotoxicity profile (Table 2). 

**Figure 4:**
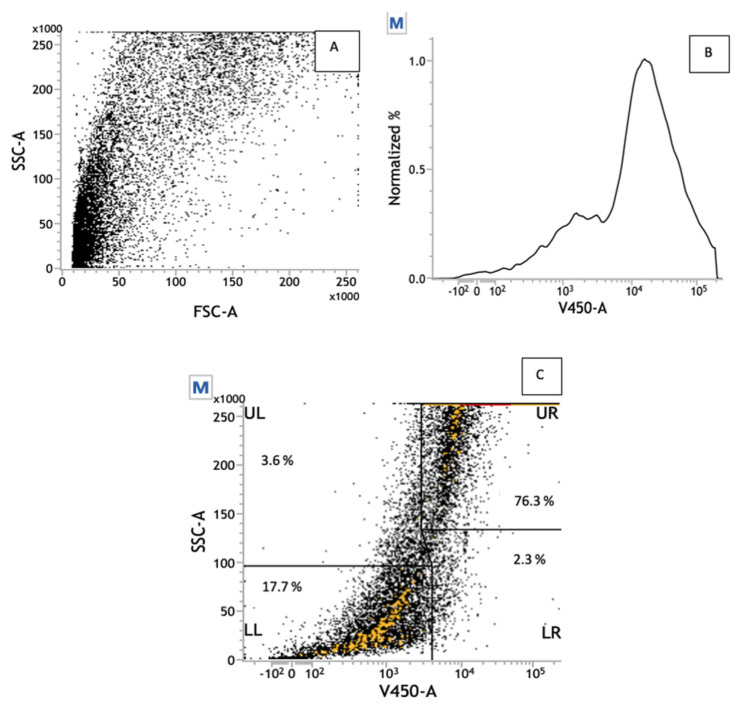
Flow cytometry panels of A549 cells treated with SL-IONPs-Ac for 48 h (iron concentration of 3.8 µg·mL^−1^), obtained from a count of 50,000 events. (**A**) Dot plot of SSC vs. FSC. (**B**) Normalized (%) histogram of V450-A, in which 2 different populations can be identified with intensity of emission at 10^3^ and 10^4^. (**C**) Gated dot plot of SSC vs. V450-A. SSC = side scatter; FSC = forward scatter.

Because the highest cell death percent was obtained after treatment with the highest dose, such concentration was chosen to be further investigated in this study. Thus, Figure 4A shows the flow cytometry panel (side scatter versus forward scatter—SSC vs. FSC), wherein the SSC and the FSC are associated with the cells’ granulometry parameters and to the cell’s diameter of the cells treated with SL-IONP-Ac at the 3.8 µg·mL^−1^ concentration. This figure reveals a wide distribution of sizes and variable internal granulometry, which indicates the presence of cells with different morphological characteristics, such as living cells, dead cells, or even cellular debris. This hypothesis can be correlated to the high number of dead cells (Figure 4B) on which was observed a characteristic peak with high intensity degree in 10^4^, in contrast to living cells that showed a peak of low intensity (10^3^) as presented in Figure 3A.

Based on the intensity of V450-A emissions, two main cell populations can be identified, as showed in the Figure 4C: (i) 76.3% of high fluorescence level, suggesting dead cells, and (ii) 17.7% of low intensity, indicative of living cells. It is important to notice that the IONPs-Ac were not able to promote any cytotoxic effect, whereas its delivery by a liposomal formulation, the SL-IONPs-Ac, was responsible for a remarkable reduction in the A549 tumor cells’ viability. This can be associated with the presence of iron oxide nanoparticles, which can be carried by liposomes to the cytosol of A549 cells, modifying, significantly, the exposition mode of the nanoparticles to the tumor cells.

**Figure 5:**
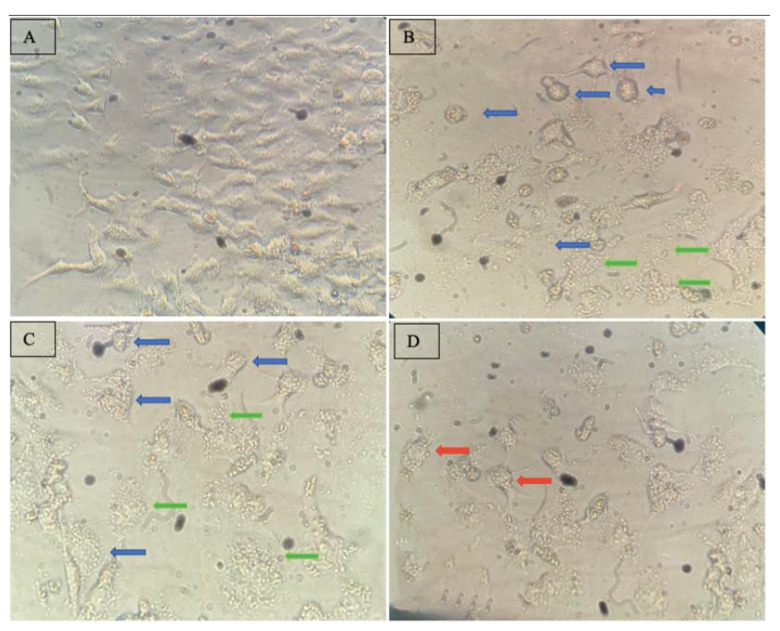
Micrographs of A549 cells cytotoxicity study (amplification of 900×). (**A**) Negative control (cells treated with NaCl solution (0.9% (*w*/*v*)). (**B**–**D**) Cells treated with SL-IONPs-Ac (3.8 µg·mL^−1^ of iron) revealing cells in possible apoptosis process (blue arrows), blebbing rounding up of the dead cells (green arrows), and possible autophagy processes (red arrows).

In addition to the flow cytometry analyses, the morphological evaluation of treated A549 tumor cells may corroborate with the obtained data from the flow cytometry study (Figure 4). In this regard, A549 cells treated with NaCl solution (0.9% (*w*/*v*)), taken as a negative control, showed a well-adherent and pebble-like growth; long fusiform shape; low quantity of cytoplasmic granules; and a clear cell boundary (Figure 5A). On the other hand, the morphology of the A549 cells treated with SL-IONPs-Ac (iron concentration of 3.8 µg·mL^−1^) for 48 h presented a completely different morphology (Figure 5B–D).

In fact, after the treatment, A549 cells presented relevant modification in their shape, assuming a circular form (blue arrows), which can be correlated to apoptotic or ferroptotic process, which is dose-dependent, as it was observed in the present study [32,33]. In addition, the presence of the cytoplasmic granules, blebs, and blebbing rounding up the dead cell (green arrows), viewed at Figure 5C,D, which are typical features of the apoptosis process, reinforce this hypothesis. However, additional tests (e. g. Western blot analysis) should be conducted to further investigate and to validate these observations.

Furthermore, possible autophagy processes were also identified (red arrows) in a low extension, compared to the suggestive apoptotic process. Therefore, based on the present results, it is possible to suggest that the cytotoxicity of SL-IONPs-Ac may be associated with the apoptosis induction.

### 3.5. Cell Death Mechanism Evaluation

Taking together, the morphological characteristics of the A549 cells treated with SL-IONPs-Ac by light microscopy allow us to suggest that their cytotoxicity may be related to the apoptosis or ferroptosis induction. Indeed, these phenomena have generated morphological features. The cells, during the ferroptosis process, present a lack of rupture and blebbing of the plasma membrane with normal nuclear size and lack of chromatin condensation. On the other hand, cells in the apoptotic process display plasma membrane blebbing, rounding up of the cell cytoplasm, reduction of the nuclear volume, and chromatin condensation [34].

**Figure 6:**
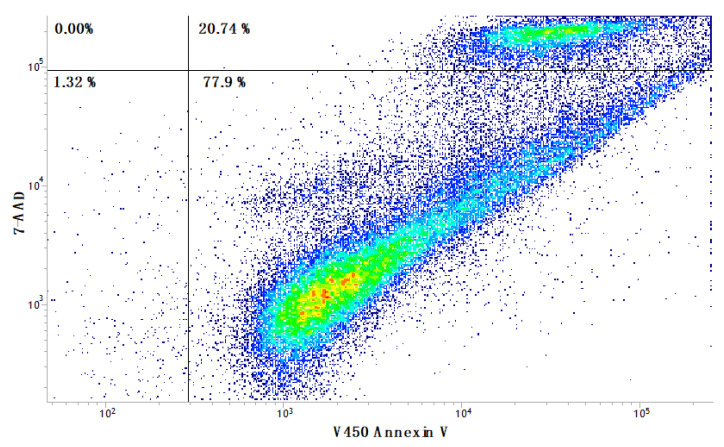
Flow cytometry panel of A549 cells treated with SL-IONPs-Ac for 48 h (iron concentration of 3.8 µg·mL^−1^): density plot of 7-AAD vs. V450 annexin-V. Data obtained from a count of 50,000 events.

Because the morphological investigation conducted in this study revealed the coexistence of different dead cell mechanisms that occurred in different proportions, an additional evaluation by flow cytometry was conducted in A549 tumor cells treated with SL-IONPs-Ac (3.8 µg·mL^−1^) for 48 h. In fact, the results suggest that the SL-IONPs-Ac were able to promote the apoptosis induction in A549 cells (Figure 6). The data available in the lower right quadrant of Figure 6 reveal a significant number of cells in early apoptosis or ferroptosis, affecting 77.94% of the tested population. Since both processes, ferroptosis and early apoptosis, are characterized by the lack of rupture of membrane, their individual contribution was determined by this method. In addition, the upper right quadrant shows A549 cells in late apoptosis or already dead, representing 20.74% of cell population. The lower left quadrant, which represents the living cells, reaches only 1.32% of the total.

This data ensures the apoptotic potential of the SL-IONPs-Ac system. However, it did not specify how this liposomal system can trigger the apoptosis process in the A549 cells. Indeed, this cell death process is complex and can be triggered by several phenomena, such as (i) oxidative stress, which leads to mitochondrial dysfunction; (ii) caspase-activation; (iii) intracellular signaling pathway, which leads to apoptosis induction; or even by (iv) mitochondrial damage, which facilitates the cytochrome C release to cytosol, leading to apoptosis [35].

Accordingly, in order to investigate possible mitochondrial changes in A549 cells treated with SL-IONPs-Ac (3.8 µg·mL^−1^) for 48 h, the mitochondrial membrane potential was evaluated using the tetramethyl–rhodamine ethyl ester (TRME). TRME allows us to evaluate the mitochondria membrane potential (∆Ψ_m_), because it can become depolarized in cells that are undergoing apoptosis, oxidative stress, necrosis, and other processes that affect the mitochondrial ions balance. Indeed, TMRE is readily sequestered by the active mitochondria of healthy cells due to the ΔΨ_m_. Therefore, cells in apoptosis/necrosis process, mediated by relevant oxidative, display loss of ΔΨ_m_ and do not accumulate relevant amounts of TMRE dye. Accordingly, the low TMRE fluorescence level of the treated cells can indicate a loss of ΔΨ_m_, ensuring the apoptosis process.

**Figure 7:**
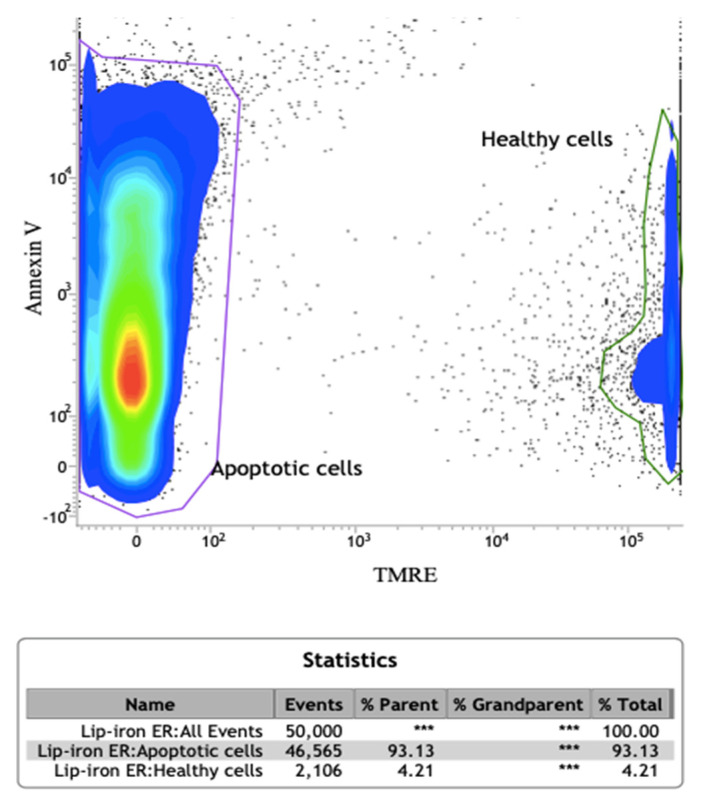
Flow cytometry panel of A549 cells treated for 48 h with SL-IONPs-Ac (iron concentration of 3.8 µg·mL^−1^): contour plot of Annexin-V vs. tetramethyl–rhodamine ethyl ester (TRME) revealing apoptotic/ferroptotic and healthy cells population. Data obtained from a count of 50,000 events. ***: data is unavailable in this specific test.

The results of this study revealed two different populations of cells (Figure 7): (i) cells that have a significant reduction in the ΔΨ_m_, probably due to induction of ferroptosis or apoptosis processes (93.13%), and (ii) healthy cells (4.21%). These results confirm that not only the tested tumor cells were susceptible to the SL-IONPs-Ac but also that this system induces the cell death by an apoptotic pathway triggered by the mitochondrial dysfunction.

In general, the cells may die from accidental cell death (ACD) or regulated cell death (RCD), which represents an essential mechanism closely related to normal cellular metabolism, tissue development, and homeostasis [36,37]. Apoptosis, necroptosis, autophagy, ferroptosis, and pyroptosis are processes related to RCD. It is important to highlight that ferroptosis is an emerging term that has been used to describe the cell death process due to the intracellular accumulation of high amounts of iron, which can occur specifically in tumor cells [38]. Furthermore, apoptosis and ferroptosis share the same feature regarding the ΔΨ_m_, wherein both are characterized by the dissipation and loss of ΔΨ_m_ [34,39]. However, ferroptosis was first observed in 2012 as a novel cell death process, observed in tumor cells [38].

Recent researches have correlated the ferroptosis process to the TP53 gene, which, at the end, reveals a possible role of the P53 protein in the regulation of the ferroptosis process [40,41]. Since the TP53 tumor suppressor is the most frequently mutated gene in human cancers, a pivotal relationship between ferroptosis, p53 protein, and tumor cells can be established and can be used to help understand the selective behavior observed in this study [42,43].

In 1997, Mukhopadhyay and Roth conducted an interesting study in which A549 cells were induced to apoptosis after the activation of the wild type p53 by methoxy–estradiol. In this study, the authors detected a high level of wild type p53 in A549 cells undergoing apoptosis [44]. Other recent studies have evidenced that reactive oxygen species (ROS) can upstream the effects of p53 in some model systems, while in others studies, ROS production can downstream the effects of p53 activation, revealing a dual important relationship between p53 and intracellular ROS level [45,46].

Considering the context herein discussed, it was possible to note that SL-IONPs-Ac system was able to induce cell death in A549 tumor cells. Therefore, it could be hypothesized that these ferri–liposomes were able to induce apoptosis/ferroptosis from the release of IONPs-Ac in the cells’ cytosol, promoting mitochondrial dysfunction that may lead to the apoptosis/ferroptosis.

### 3.6. Selectiveness Cytotoxicity Study

The main disadvantage of drugs and medicines used for antineoplastic treatment is their poor selectiveness, which impairs their use due to several side effects that are reported from antitumor drugs. Therefore, antitumor therapy uses different approaches for targeting tumor cells. However, despite the plethora of nanotechnological devices that have been developed, the target to specific tumor cells remains a challenger.

During the pre-formulations studies to produce new medicines in the cancer therapy field, it is important to evaluate not only its effectiveness but also the safety and/or selectiveness of the developed system. To this regard, to evaluate the ability of the SL-IONPs-Ac ferri–liposomes formulation to selectively kill tumor cells instead of normal cells, HEK-293 cells were treated with SL-IONPs-Ac at 3.8 µg·mL^−1^ of iron oxide for 48 h.

**Figure 8:**
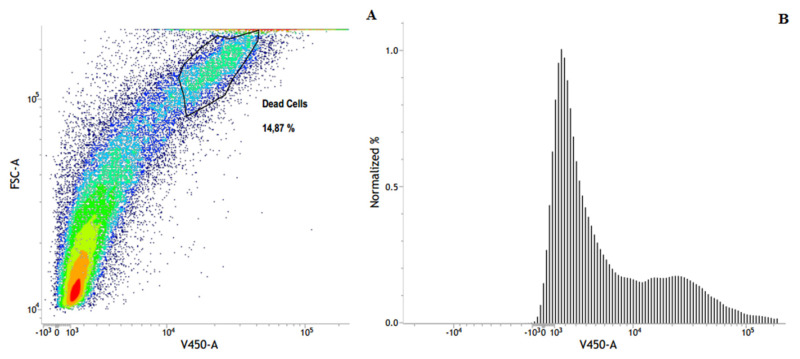
Flow cytometry panels of HEK-293 cells treated for 48 h with SL-IONPs-Ac (iron concentration of 3.8 µg·mL^−1^). (**A**) Contour plot of FSC vs. V450-A in which the percentage of dead cells was quantified as 14.87%. (**B**) Density plot of SSC vs. V450-A in which the healthy population can be identified, reaching 85.13%. Data obtained from a count of 50,000 events.

The results revealed that an important percentage of cells remained viable (Figure 8), even after being submitted to the same treatment protocol in which a significant number of A549 cells were conducted to the death (Figure 7). Indeed, although the flow cytometry panel of treated HEK-293 cells (contour plot of FSC vs. V450) revealed the presence of two different populations of cells, only 14.87% was associated with dead ones (Figure 8A), while 85.13% indicates the percentage of healthy cells.

The safety and the potentiality of nanostructured systems intended for antineoplastic treatment at concentrations lower than the pure drug has been evidenced by several authors, such as Amaral-Machado et al. (2016) and Zatta et al. (2018). Amaral-Machado et al. (2016) developed an emulsified nanostructured system able to induce melanoma cell death without promoting expressive cytotoxicity in healthy fibroblasts [47]. Zata et al. (2018) showed that nanoparticles with 5-fluorouracil were able to promote the death of different metastatic melanoma cells in concentrations up to 4.3-fold lower than the pure 5-fluorouracil, suggesting the safety of the developed system [48].

The aforementioned studies reinforce not only the importance of investigations regarding the safety/selectiveness of novel nanostructured systems in antineoplastic therapy but also corroborate the findings herein obtained, in which the selective cytotoxicity induction of tumor cells from SL-IONPs-Ac was observed. Overall, the results here presented showed that the liposomal system was able to carry the IONPs-Ac, presenting suitable physiochemical features, desirable stability and, also, a remarkable specific cytotoxic effect on lung adenocarcinoma cells.

## 4. Conclusions

The developed ferri–liposomes formulation revealed important storage stability and displayed a relevant selective cytotoxic profile against human lung adenocarcinoma cells, in which the apoptosis/ferroptosis were attributed as the possible main mechanisms of death. On the other hand, citric acid–iron oxide nanoparticles, alone, appeared to be practically innocuous to the same cells under the same treatment conditions. It could be suggested from the present study that ferri–liposomes enabled a significant increase in the iron oxide nanoparticles internalization in A549 cells that culminated in the imbalance of the ΔΨ_m_ and, by consequence, induced cells death. Another interesting result was achieved when HEK-293 cells, normal human embryonic kidney cells, were submitted to the same treatment. The tested samples did not induce any significant cytotoxicity effect, revealing an important antitumor selectiveness. The search for more selective agents against cancer must be the focus for the development of better treatments that, fundamentally, can provide a better quality of life to the patients. Accordingly, further studies may reveal more details regarding the biochemical pathways involved in the cytotoxic activity of the ferri–liposomes in tumor cells. Therefore, the overall results open a new perspective for liposome-based therapy to be used for lung cancer. This carrier revealed to be a potential antitumor nanosystem with remarkable selectivity against lung adenocarcinoma cells.

## Figures and Tables

**Table 1:** Size distribution results of IONPs-Ac and SL-IONPs-Ac samples.

Sample	Average Size (nm)	PdI
IONPs-AC	23.2 ± 1.1	0.31 ± 0.03
SL-IONPs-AC	189.2 ± 12.2	0.08 ± 0.01

**Table 2:** Cytotoxicity evaluation of different SL-IONPs-Ac concentrations.

SL-IONP-Ac Concentration (µg·mL^−1^)	Cell Death (%)
3.8	75.3 ± 6.4
1.9	58.3 ± 5.3
0.9	31.4 ± 2.7

## Data Availability

Not applicable.
